# The new survivors and a new era for trauma research

**DOI:** 10.1371/journal.pmed.1002354

**Published:** 2017-07-25

**Authors:** Karim Brohi, Martin Schreiber

**Affiliations:** 1 Centre for Trauma Sciences, Queen Mary University of London, London, United Kingdom; 2 Department of Surgery, Oregon Health & Science University, Portland, Oregon, United States of America

## Abstract

Karim Brohi and Martin Schreiber, Guest Editors of the Special Issue on Trauma, describe a new era in exploration of the biology of injury response and translation of new opportunities into clinical practice.

Trauma—physical injury to the body and its sequelae—is one of the greatest killers of humanity, taking young lives and inducing suffering and disability worldwide. Population, societal, and climate change are all increasing the incidence and severity of trauma. Despite its high global impact, trauma remains low on governmental priorities, a side concern for funders and of only passing interest to most scientists. The opportunities for trauma research are great, with the potential to create new survivors, who live stronger, healthier lives, with reduced health care and societal burdens. Achieving these outcomes requires innovative diagnostics, therapeutics, and devices, applicable to addressing the global burden of injury in a variety of settings. As a disease of damage, shock, and sterile inflammation, with a known and timed insult, trauma can be a model for scientific discoveries in infection, immunity, and inflammatory conditions more generally. We are on the cusp of a new era of trauma research, with research infrastructures and collaborations that can explore the biology of the human response to injury and effectively translate new opportunities into clinical practice.

The trauma community has always been active in research, but until the turn of this century lacked the infrastructure that had been developed in other areas of study such as cardiovascular disease and cancer. Research relied on individual investigators and their laboratories, and outputs were difficult to externally validate. A lack of an ethical framework for enrolling incapacitated patients meant early phase IIa and IIb studies were often impossible. Large-scale phase III or IV studies were exceedingly rare due to lack of collaborative research networks. Thus, trauma clinical practice was often based on relatively weak trauma studies and on findings from other conditions, which were easier to study but not actually comparable. As a result, the research field was largely stagnant, with few dedicated researchers, disengaged funders, and management paradigms that were incorrect and resulted in potentially avoidable deaths.

The last 2 decades, in contrast, have seen an upsurge in trauma research capability and capacity. There have been several drivers for this, including opportunities identified by pharmaceutical and diagnostics industries [1]; innovation driven by military demands in global conflict arenas [2]; and the coalescence of civilian multicentre research networks [3,4,5]. The resulting critical mass of activity has highlighted the need for ethical frameworks for patients who lack capacity and regulatory frameworks that support the translational research process in emergency care.

Research into haemostasis and the prevention of deaths due to bleeding has been at the centre of this activity. Industry-led clinical trials of recombinant factor VIIa in the 1990s were fueled by innovation in conflict settings and conducted by civilian trauma centres worldwide [1]. Although the trials did not show a survival benefit for the study drug, they showcased the huge global demand for trauma care solutions, demonstrated that effective, efficient multicentre civilian research was possible, and left a legacy of research collaborations that could evolve into future networks. Investigation of the human haemostatic response elucidated the specific pathophysiology of trauma-induced coagulopathy, which in turn led to an explosion of innovation in diagnostics, devices, and therapeutics [6]. As a result, resuscitation has rapidly changed from a perfusion-centred approach based on nontrauma research to a haemostatic-centred approach grounded in trauma biology [7], with attendant reductions in mortality and morbidity worldwide.

This work has shown that high quality trauma research is both possible and impactful and has driven the development of translational and clinical research infrastructures that, while still nascent, can match those in more established areas of disease research. We now have the opportunity to leverage these platforms to advance other areas of trauma research. Understanding the acute response to injury and its dysfunctional aspects will be central to future developments and now requires basic scientists to focus their expertise on the pathobiology of trauma. Scientists will need to directly engage with clinical researchers to explore the activation and evolution of the response to injury, even in the first minutes after injury at the incident scenes. We need new diagnostics, suitable for these environments that will allow us to identify relevant phenotypes and deliver personalised medicine in tight timeframes. Large multicentre and multinational research databases will be required (and, in part, already exist) to understand how hyperacute phenotypes and specific interventions track to short-, medium-, and long-term health outcomes. We will need engagement from bioinformaticians and data scientists to link datasets and generate mechanistic hypotheses and new targets for pharmaceutical intervention. In this way, we can ensure that more people survive with better health outcomes from severe physical damage, extreme shock and ischaemia, and especially from traumatic brain injury. These changes are likely to usher in a new era of trauma resuscitation, incorporating cell- and organ-protective modalities into a broader strategic approach to preserving physiological competence after injury.

Opportunities exist not just for avoiding death but for returning survivors to full health and function. The global demand for solutions in reconstruction, regeneration, and rehabilitation across the patient’s journey is immense. The opportunities offered by bioengineering, regenerative medicine, robotics, and digital technologies are potentially transformative. Innovators in academia and industry can now collaborate and capitalise on more defined translational pathways, clearer regulatory frameworks, and access to patients through clinical research networks. This work is also likely to benefit a broad range of nontrauma conditions with components of tissue loss, healing, and loss of function.

Research driven by clinical imperative has delivered dramatic developments in trauma care over the past 2 decades. These advances have ushered in new understandings and new paradigms of trauma management. This research landscape, of translational infrastructure and clinical research networks, is the new platform on which to engage with scientists and innovators across academia and industry. With a clear strategy and robust support, we can greatly increase the chances not only that the injured survive but that survivors have an opportunity to resume meaningful lives.
